# The problem of *Mycobacterium abscessus* complex: multi-drug resistance, bacteriophage susceptibility and potential healthcare transmission

**DOI:** 10.1016/j.cmi.2023.06.026

**Published:** 2023-06-24

**Authors:** Rebekah M. Dedrick, Lawrence Abad, Nathaniel Storey, Ari M. Kaganovsky, Bailey E. Smith, Haley A. Aull, Madison Cristinziano, Anna Morkowska, Saraswathi Murthy, Michael R. Loebinger, Graham F. Hatfull, Giovanni Satta

**Affiliations:** 1) Department of Biological Sciences, University of Pittsburgh, Pittsburgh, PA, USA; 2) Department of Microbiology, Virology and Infection Control, Great Ormond Street Hospital for Children NHS Foundation Trust, London, UK; 3) Microbiology, North West London Pathology, London, UK; 4) Royal Brompton and Harefield Hospitals, Guys and St Thomas’s NHS Foundation Trust, London, UK; 5) National Heart and Lung Institute, Imperial College London, London, UK; 6) Centre for Clinical Microbiology, University College London, London, UK; 7) Infection Division, University College London Hospitals NHS Foundation Trust, London, UK

**Keywords:** Bacteriophages, Multi-drug resistance, *Mycobacterium abscessus* complex, Next-generation sequencing, Prophages

## Abstract

**Objectives:**

*Mycobacterium abscessus* complex is responsible for 2.6–13.0% of all non-tuberculous mycobacterial pulmonary infections and these are notoriously difficult to treat due to the complex regimens required, drug resistance and adverse effects. Hence, bacteriophages have been considered in clinical practice as an additional treatment option. Here, we evaluated antibiotic and phage susceptibility profiles of *M. abscessus* clinical isolates. Whole-genome sequencing (WGS) revealed the phylogenetic relationships, dominant circulating clones (DCCs), the likelihood of patient-to-patient transmission and the presence of prophages.

**Methods:**

Antibiotic susceptibility testing was performed using CLSI breakpoints (*n* = 95), and plaque assays were used for phage susceptibility testing (subset of *n* = 88, 35 rough and 53 smooth morphology). WGS was completed using the Illumina platform and analysed using Snippy/snp-dists and Discovery and Extraction of Phages Tool (DEPhT).

**Results:**

Amikacin and Tigecycline were the most active drugs (with 2 strains resistant to amikacin, and one strain with Tigecycline MIC of 4 μg/mL). Most strains were resistant to all other drugs tested, with Linezolid and Imipenem showing the least resistance, at 38% (36/95) and 55% (52/95), respectively. Rough colony morphotype strains were more phage-susceptible than smooth strains (77%—27/35 versus 48%—25/53 in the plaque assays, but smooth strains are not killed efficiently by those phages in liquid infection assay). We have also identified 100 resident prophages, some of which were propagated lytically. DCC1 (20%—18/90) and DCC4 (22%—20/90) were observed to be the major clones and WGS identified 6 events of possible patient-to-patient transmission.

**Discussion:**

Many strains of *M. abscessus* complex are intrinsically resistant to available antibiotics and bacteriophages represent an alternative therapeutic option, but only for strains with rough morphology. Further studies are needed to elucidate the role of hospital-borne *M. abscessus* transmission.

## Introduction

Non-tuberculous mycobacteria (NTM) are mycobacterial species other than the *Mycobacterium tuberculosis complex and Mycobacterium leprae*. They are ubiquitous in the environment and are often isolated from water, soil and hospital wards [1]. With increasing numbers of immunocompromised patients, as well as patients with cystic fibrosis (CF) and chronic lung disorders, the role of NTM as a cause of human disease has become apparent, with various reports indicating a worldwide increase [2,3]. In particular, the *Mycobacterium abscessus* complex comprises a group of rapidly growing, multi-drug-resistant, NTM that are responsible for a wide spectrum of respiratory infections, skin and soft tissue diseases, ocular and other infections. The *M. abscessus* complex is differentiated into three subspecies: *M. abscessus* subsp. *abscessus*, *M. abscessus* subsp. *massiliense*, and *M. abscessus* subsp. *bolletii* [4]. In the United States, *M. abscessus* complex infections are secondary only to *Mycobacterium avium* complex infections, compromising 2.6–13.0% of all mycobacterial pulmonary infections [5]. There is also an ongoing debate regarding the potential impact of direct patient-to-patient transmission of *M. abscessus* in high-risk group patients such as those with CF and the number of single-nucleotide polymorphisms (SNPs) needed to confirm direct spreading [6,7].

Current treatment guidelines [8,9] are based on limited data and often suggest administration of macrolide-based therapy in combination with intravenous antibiotics, with substantial side effects for patients [9,10].

Bacteriophages (or phages) have been proposed (and used in Eastern Europe) for the treatment of bacterial infections, but their clinical use in the Western world is generally on case-by-case, under compassionate use authorization [11]. More recently, their clinical utility has been re-discovered because of the rise of multi-drug resistance and their potential use in clinical practice as an additional treatment option [12]. Phage activity has been successfully demonstrated against different mycobacterial species, including *M. abscessus* and *M. tuberculosis* [13,14].

The first successful clinical case of phage treatment against drug-resistant *M. abscessus* was in a 15-year-old patient with CF with double lung transplant and disseminated infection [15]. In a 20-patient consecutive case series of mycobacterial infections (*abscessus, chelonae*, Bacillus Calmette Guerin (BCG), and *avium*) treated with mycobacteriophages, 11 (55%) had a favourable response, 4 showed no clinical improvement, and outcomes for 5 patients were inconclusive [16].

Treating patients with *M. abscessus* infections with phages is challenging because there is high strain variability in the phage infection profiles and these are not predictable genomically [14]. Colony morphotype is a key factor, and ~75% of rough strains are infected and killed by at least one of a small panel of phages tested; some smooth colony morphotype strains are efficiently infected by some phages as assessed by plaque formation, but none are efficiently killed in challenge assays [14].

The primary aim of this project was to assess the extent of drug resistance in *M. abscessus* clinical isolates from two tertiary referral centres in London. The secondary aim was to test those resistant isolates against some of the most therapeutically useful phages. Whole-genome sequencing (WGS) has also been performed on all clinical strains to determine the dominant circulating clones (DCCs), potential transmission and the presence of prophages.

## Methods

### Bacterial strains

All *M. abscessus* culture positive clinical isolates were collected from the microbiology diagnostic laboratory in two referral centres in London over a 2-year period (2018–2019). Identification to species level was initially performed using the GenoType NTM (Hain Lifescience GmbH, Nehren, Germany) and later confirmed by WGS. *M. abscessus* ATCC19977 was obtained from the American Type Culture Collection. *M. abscessus* GD41 [16], BWH-C [17] and *M. smegmatis* mc^2^155 [18] have been previously reported. All *M. abscessus* strains were grown on Middlebrook 7H10 agar (Difco) supplemented with oleic acid-albumin-dextrose-catalase and 1 mM CaCl_2_ and incubated at 37°C for 5–7 days. Liquid cultures were grown in Middlebrook 7H9 (Difco) with oleic acid-albumin-dextrose-catalase and 1 mM CaCl_2_ inoculated from a single colony and incubated at 37°C for 5–7 days with shaking. For plaque assays, *M. abscessus* cultures were sonicated [17].

### Antibiotic susceptibility testing

Susceptibility testing was performed using the commercially available kit Sensititre^®^ RAPMYCO (ThermoFisher Scientific, Waltham, USA) and CLSI breakpoints (CLSI M24, 3rd edition) [19] against various antibiotics (Table 1). Plates were incubated at 37°C with first reading at 5 and 7 days and further incubated for an additional 7 days (total of 14) for the detection of inducible Clarithromycin resistance [20].

### Phage susceptibility testing

Top agar overlays of *M. abscessus* cultures were created by mixing 1 mL saturated culture, 4 mL 7H9, and 7.5 mL melted Middlebrook Top Agar (MBTA), and subsequently spread over with 7H10 bottom agar with 1 mM CaCl_2_, 50 μg/mL Carbenicillin, and 20 μg/mL Cycloheximide in 150 × 15 mm petri dishes. Eight 10-fold serial dilutions of phage lysates (Table 1) were spotted onto the top agar overlays of control strains—*M. smegmatis* mc^2^155, *M. abscessus* GD41, *M. abscessus* ATCC19977, *M. abscessus* BWHC—and each clinical isolate, and incubated at 37°C for 4–5 days.

### WGS

DNA was extracted using the DNeasy^®^ Blood and Tissue Kit (Qiagen, Hilden, Germany), and sequencing performed using the Illumina NextSeq 500 (Illumina, San Diego, USA). SNPs were called from the short read sequencing data with Snippy version 4.3.6 (freely available on GitHub) using the sequence of *Mycobacteroides abscessus* strain GZ002 (Accession: CP034181.1) as a reference. A pairwise SNP distance matrix was generated snp-dists version 0.8.2 (also available on GitHub).

### Phylogenetic trees

A whole-genome alignment was created by aligning assemblies to the reference strain ATCC19977 with Parsnp [21] and Gubbins. IQ-TREE (http://www.iqtree.org/) was run on the alignment using the general time-reversible model with fast search enabled. The produced phylogenetic tree was visualized using FigTree and iTOL [22]. DCCs were identified by analysing genetic distances to reference strains as defined previously [7,23,24].

### Prophage identification and propagation

DEPhT [25] was used to identify and extract complete prophages, and incomplete prophage sequences spanning contig gaps were identified using SPLICE (to be described elsewhere) in addition to DEPhT. Gene functions were annotated using Hidden Markov Models of mycobacteriophage protein sequences clusters. Lytic propagation of spontaneously released prophages and PCR characterization was performed as previously described [14].

### Statistical analysis

Chi-square tests were used by comparing two different rates, with a degree of freedom of one.

## Results

### M. abscessus strain phylogenies

One hundred *M. abscessus* isolates were collected from 86 patients with either soft tissue or respiratory infections. These isolates included 14 repeat samples from the same patient taken at least 6 months apart. Antibiotic susceptibility information was collected for 95 samples, because of 5 strains being removed from the analysis after being identified as not *M. abscessus* on WGS (probably because of an original misidentification with the GenoType probe), whereas genomic information was considered for 90 isolates (because of sequencing quality issues and the removal of the misidentified strains). In terms of colony morphology, the strains were recorded as either smooth (60%—57/95) or rough (40%—38/95). On the basis of the available sequencing data, the majority of strains were identified as *M. abscessus subsp. abscessus* (72%—65/90), followed by *M. abscessus subsp. massiliense* (22%—20/90), and 5 were *M. abscessus subsp. bolletii*. Phage susceptibility was performed on 88 strains in total, because of some strains failing to grow again and the removal of some of the repeat strains.

**Fig. 1.**
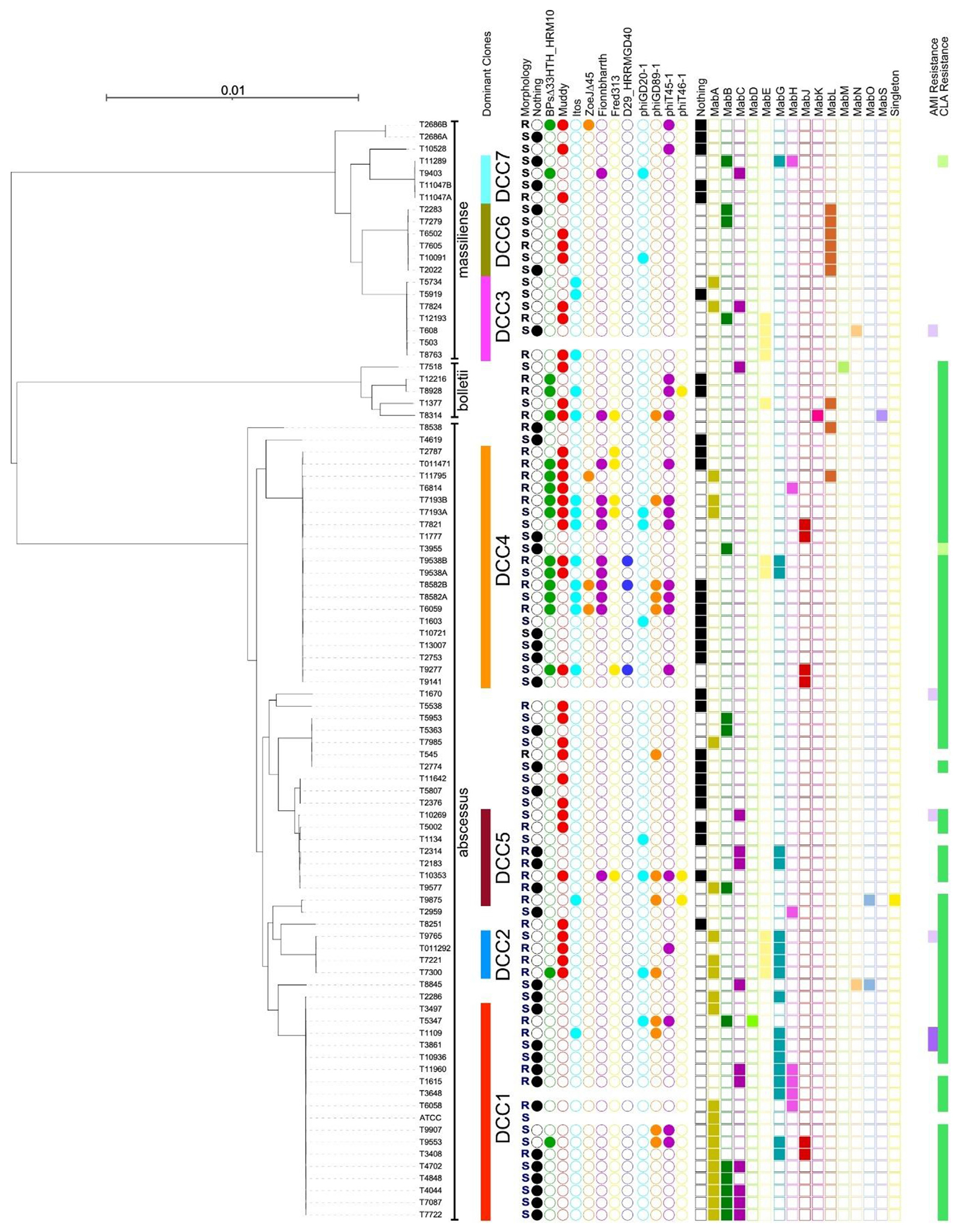
Phylogeny and phage characteristics of *M. abscessus* strains. The phylogeny of 90 *M. abscessus* strains with reference strains is shown and each is assigned to dominant circulating clones (DCC1–DDC7) as defined previously [7]. Strains chosen to represent previously characterized dominant circulating clones are the following: ATCC19977 for DCC1, GD54 for DCC2, G220 and JHN_AB_0023_1 for DCC3, 976 for DCC4, 1100 for DCC5, A47 for DCC6 and FLAC047 for DCC7 as performed in previous studies [24]. Strain subspecies designations are shown (*massiliense, bolletii, abscessus*). The colony morphotype of each strain is indicated (R = rough, S = smooth), together with their susceptibilities to infection by 7 *M. smegmatis* phages and 4 lytically grown phages derived from *M. abscessus* prophages and described previously [17] (coloured circles). Filled circles indicate either no phage infection (black) or an efficiency of plating relative to *M. smegmatis* of greater than 10^−2^. Some strains did not propagate well and their susceptibilities are not shown. The prophage content of each strain is shown by coloured squares with filled squares noting the presence of a prophage grouped within clusters (MabA, MabB, etc., or singleton) as indicated above. The AMI and CLA resistance profiles are indicated at the extreme right. Purple indicates resistance to Amikacin while green indicates resistance to Clarithromycin. Light purple and light green indicate intermediate resistance to Amikacin and Clarithromycin, respectively. AMI, Amikacin; CLA, Clarithromycin; *M. abscessus, Mycobacterium abscessus; M. smegmatis, Mycobacterium smegmatis*.

Only 69 of the samples were able to be assigned to DCCs as defined previously [7] (Fig. 1). All 7 DCCs of *M. abscessus* were represented, with most isolates in DCC4 (22%—20/90), followed by DDC1 (20%—18/90), DCC5 (11%—10/90), DCC3 (8%—7/90), DCC6 (7%—6/90), DCC2 (4%—4/90), DCC7 (4%—4/90) and 21 strains with no DCC. Whole-genome SNP analysis identified isolates with ≤10 SNPs difference (*n* = 8 pairs) and ≤20 SNPs (*n* = 5 pairs). Among the isolates with ≤10 SNPs, only 2 pairs represented repeat samples from the same patients at different time points, with 5 and 10 SNPs difference. All other samples were collected from different patients during the same year, with 3 pairs having 5 SNPs difference, one 7 SNPs, one 8 SNPs and another 10 SNPs. Among the isolates with ≤20 SNPs, again 2 pairs represented repeat isolates from the same patients and the other 3 pairs were from different patients, with 18, 19 and 20 SNPs difference, respectively. All the above isolates also belonged to DCC1, apart from the pair with 18 SNPs differences which belonged to DCC4 (Table S1).

Further bioinformatics comparison (File S4) of our samples with all the DCC1 strains (*n* = 330) used in a previous paper [24] showed most strains having a high number of SNPs difference compared with our database, with none showing a difference of <10 SNPs with any samples in our collection.

### Antibiotic susceptibility of the clinical isolates

Tigecycline and amikacin were the most active drugs. Only 2 strains were resistant to amikacin (minimum inhibitor concentration, MIC >64 μg/mL), whereas Tigecycline MICs were between 0.12 and 2 μg/mL (average: 0.61—median value: 1), with only one strain with MIC 4 μg/mL (Table 2) [26]. The majority of *M. abscessus* isolates were resistant to Clarithromycin (66%—63/95), many of which are subsp. *abscessus* and subsp. *bolletii*, although only one sample of subsp. *massiliense* showed resistance to Clarithromycin. There was high level of resistance for all other drugs tested, with Linezolid and Imipenem showing the least resistance, at 38% (36/95) and 55% (52/95), respectively (Table 1).

### Phage susceptibility testing

**Fig. 2.**
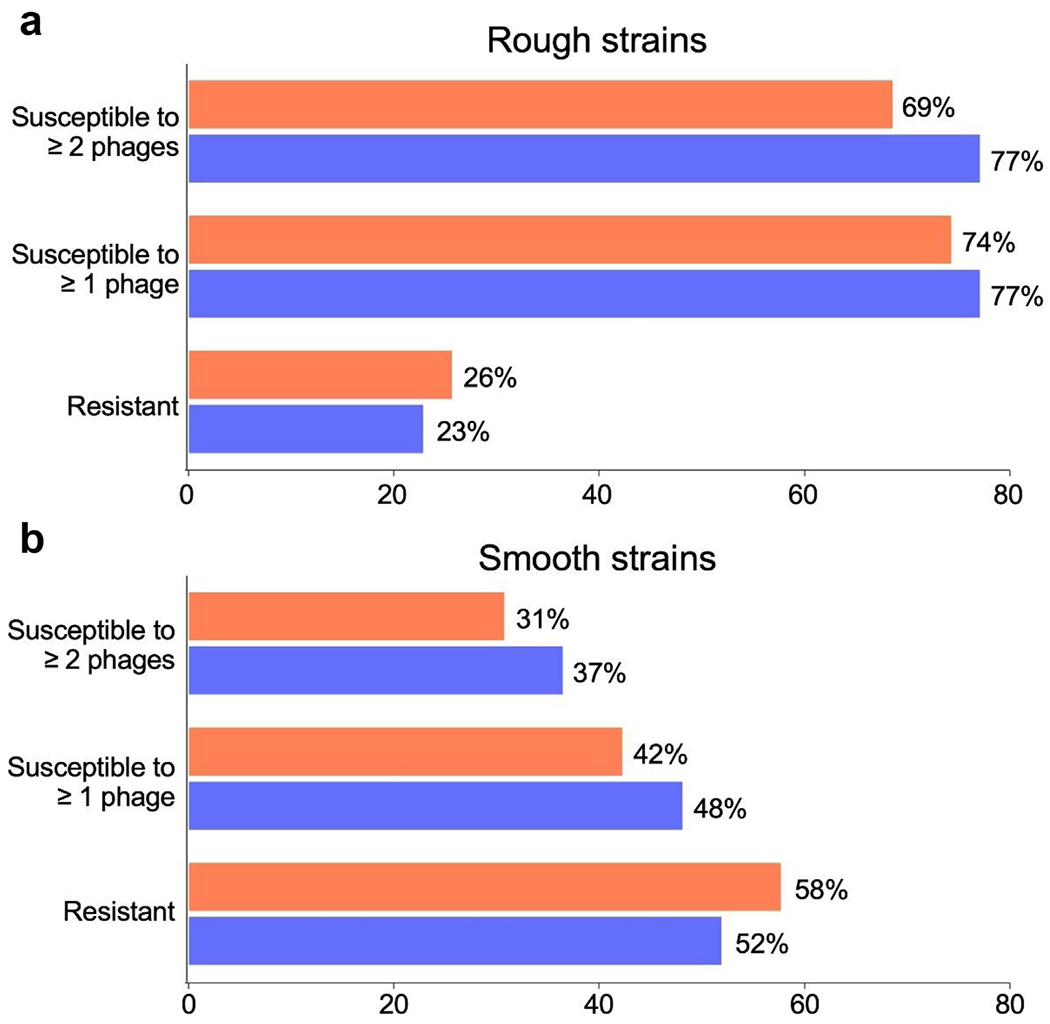
Summary of *M. abscessus* phage susceptibilities. The proportions of rough morphotype (a) or smooth morphotype strains (b) susceptible to either one or two more phages, or resistant to all phages tested are shown. The susceptibilities for just the 7 *M. smegmatis* phages tested (orange) or all 11 phages shown in Table 2 (blue) are shown. *M. abscessus, Mycobacterium abscessus; M. smegmatis, Mycobacterium smegmatis*.

Phage susceptibilities were determined as the efficiency of plating relative to a fully susceptible host, and we tested 7 therapeutic *M. smegmatis* phage candidates [16], and 4 prophage-derived phages (Figs. 1 and 2). Together, they infected 77% (27/35) of the rough colony morphotype strains (efficiency of plating >0.01), but only 48% (25/53) of the smooth strains (when considering one phage), reflecting infection patterns reported previously [14] (Figs. 1 and 2). Strikingly, phage infection profiles often vary among strains of the same DCC. For example, some strains (e.g. T13007) of DCC4 are not efficiently infected by any of the phages, but several DCC4 strains are infected by as many as seven phages, but in different combinations (Fig. 1).

### Resident prophages in M. abscessus strains

**Fig. 3.**
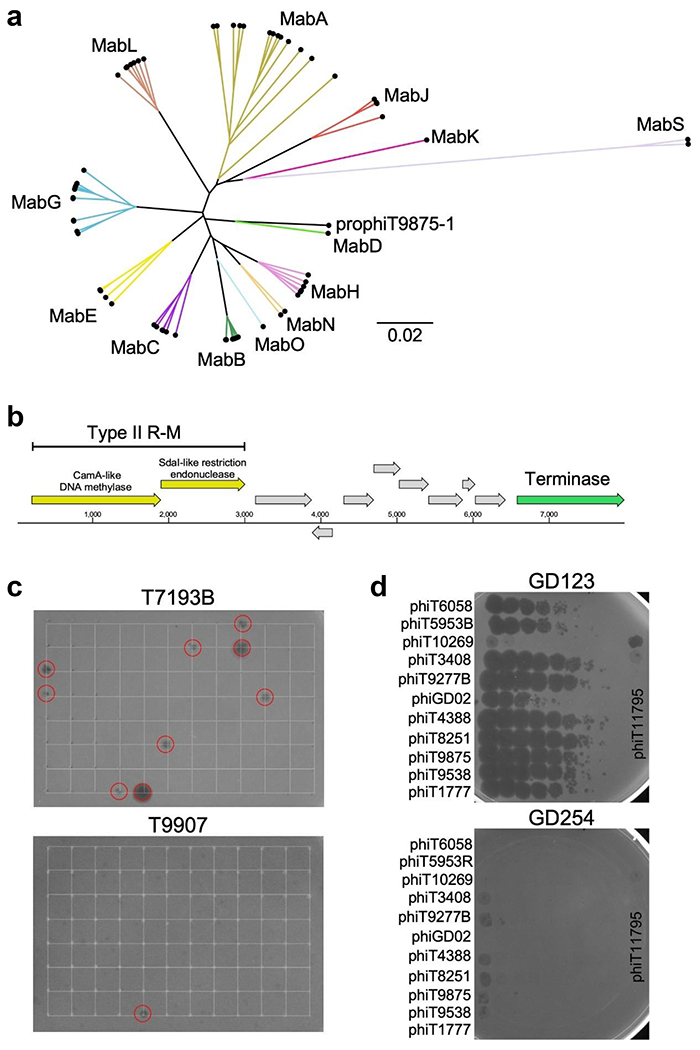
Characterization of *M. abscessus* prophages. (a) Gene content distance tree of identified prophages generated by phamnexus (available on GitHub at https://github.com/chg60/phamnexus) and the UPGMA algorithm available in Splitstree 4. The tree was illustrated with FigTree, where branches are labelled and coloured according to their designated clusters, similar to Fig. 1. ProphiT9875-1 shares little gene content with any previously identified phages although the genome shares some similarity to MabD cluster phages. (b) Illustration of the regions within prophages of T1615 and T11960, which encode a type II restriction-modification system. (c) Example solid media plates of screens for lytically growing spontaneously released phages. Lawns of *M. abscessus* strains T7193B and T9907 are shown growing on solid media, and 96 culture supernatants were spotted onto the lawns at positions indicated by the grids. Several supernatants show lytic infection (red circles), which were then purified and amplified. Strain T9907 is more typical in showing sensitivity to few or no supernatants; T719B is susceptible to a relatively high proportion of released phages and is also sensitive to five of the *M. smegmatis* phages (Fig. 1). (d) Examples of strain sensitivities to lytically growing prophages. Phage lysates were diluted and spotted onto lawns of *M. abscessus* GD123 and GD254, which illustrate the variations in phage infection profiles. Phage phiGD02 is a previously isolated control phage. *M. abscessus, Mycobacterium abscessus; M. smegmatis, Mycobacterium smegmatis*.

*Mycobacterium* prophages are known determinants of phage host range [18]. Bioinformatic analysis [25] showed that 78% (70/90) of the strains described here contain at least one prophage, and as many as 3 (Fig. 1, Table S2), and each could be assigned cluster designations based on their genomic relationships (Figs. 1 and 3(a), Table S2). Two prophages form a new Cluster MabS and one prophage (prophiT9875-1) is designated as a ‘singleton’ with no known close relatives. The two MabS prophages are notable in having many (>16) tRNA genes, polymorphic toxin-immunity systems, recET systems and predicted siphoviral morphologies. In general, the prophage constitution of the strains does not correlate closely with the phage infection profiles, suggesting that other host-encoded defences are also important. Interestingly, we note that prophages in two DCC1 rough strains (T1615 and T11960) encode type II restriction-modification systems (Fig. 3(b)), and both strains are resistant to all the phage tested (Fig. 1).

### Lytic growth of M. abscessus prophages

Most of the prophages are predicted to be intact and capable of lytic growth, although lytic propagation depends on identification of a permissive host strain. Screening of culture supernatants with a panel of potentially sensitive strains (Fig. 3(c)) enabled recovery of 24 lytically growing phages which were purified and amplified (Table S3); each is designated according to the strain they were released from (e.g. phiT9043 from strain T9043) and PCR amplification was used for preliminary cluster assignments (Table S3). Screening of these against *M. abscessus* strains showed great variation in susceptibilities, as illustrated by clinical isolates GD123 and GD254. These lytically growing phages thus represent a substantial expansion of those with potential therapeutic interest.

## Discussion

Treatment of *M. abscessus* infections remains suboptimal, with our data showing a limited availability of suitable options for patients (especially for the subsp. *abscessus*), with mostly inhaled or intravenous antimicrobials (i.e. amikacin, tigecycline, and imipenem), a high resistance rate to macrolide and poor understanding of the relationship between laboratory susceptibilities and clinical outcomes [27]. Its innate resistance to multiple antibiotics is reflected in clinical failure, with fewer than half of the patients meeting the criteria for treatment success and nearly 80% of them developing adverse events [28]. There is an important need to consider alternative antibiotics (alone and in combination for synergistic effects) especially those available *via* the oral route and more research is needed on this issue.

Considering the limited availability of active antibiotics, the therapeutic use of phages shows some potential. However, strain morphology is a key determinant and the strains tested here reveal similar infection profiles to previous reports [14]. Because it was shown previously [14] that smooth colony morphotype *M. abscessus* strains that are sensitive to some phages in plaque assays are not killed efficiently by those phages in liquid infection assays, then smooth strains (including those described here) are not therapeutic candidates. Similar discrepancies between phage infection assays have been reported for other bacterial strains including Salmonella and Staphylococcus [29,30]. In contrast, we predict that most of the rough strains reported here that are sensitive to one or more phages could potentially be considered as therapeutic candidates. Interestingly, ~78% of the strains carry at least one prophage and these likely contribute to the phage infection profiles, as illustrated by the finding of prophage-encoded type II R-M systems (Fig. 3(b)). The lytic propagation of spontaneously release prophages presents a powerful expansion of the repertoire of therapeutic candidates (Table S3), although we note that these are temperate and require engineering to be obligatorily lytic for clinical use.

In addition to the laboratory and clinical challenges, *M. abscessus* also represents an infection control dilemma. Previous outbreaks have been described in lung transplant and cardiac surgery units related to hospital tap water, with environmental and clinical isolates differing by up to 7 SNPs [31]. The same cutoff of 7 SNPs was used by other authors to determine possible patient-to-patient transmission among children in an Australian CF centre [6]. However, recent data also highlight that most patients are infected with one of the circulating DCCs, indicating the presence of global transmission networks of *M. abscessus* among both CF and non-CF individuals but with unclear spread [7,23]. In our cohort, we were able to identify at least six events of potential patient-to-patient transmission, but as they belong to the same DCC1, it makes it difficult to confirm if healthcare transmission happened versus a wider community source. Further comparison with published DCC1 strains did not highlight any close relationship (<10 SNPs) with other global strains, pointing out a possible patient-to-patient or local environmental transmission (in particular, for those isolates with <7 SNPs difference). A limitation of this study is that there were no further infection control investigations looking at the opportunities for patient contacts within their clinical interaction or any environmental and water testing were performed. Other limitations include the limited number of clinical samples, the restricted location (two hospitals in London) and the limited number of antibiotics tested because of the lack of available breakpoints for interpretation.

In conclusion, infections caused by *M. abscessus* complex represent a challenging clinical problem. Our strains showed a high level of resistance providing limited therapeutically active drugs to patients. Bacteriophages may represent an interesting alternative option, but the higher resistance rate in smooth strains limits broader applicability. There are also important infection control implications and further studies are needed to address these challenges.

## Supplementary Material

Table S1

Table S3

Table S2

Dataset 1

## Figures and Tables

**Table 1:** Antibiotic susceptibility of 95 *M. abscessus* complex isolates as per CLSI breakpoints

Antibiotic	Susceptible (%)	Intermediate (%)	Resistant (%)
Amikacin	95	3	2^a^
Cefoxitin	10	71	19
Ciprofloxacin	7	16	77
Clarithromycin^b^	32	2	66^c^
Doxycycline	1	0	99
Imipenem	4	41	55
Linezolid	30	32	38
Moxifloxacin	8	10	82
Tobramycin^d^	2	3	95
TMP-SMX	25	N/A	75
Tigecycline^e^	N/A	N/A	N/A

MIC, minimum inhibitor concentration; *M. abscessus, Mycobacterium abscessus; M. chelonae, Mycobacterium chelonae*.

a Resistant isolates were retested for confirmation.

b Result after 14 d of incubation.

c 73% resistance rate if considering *M. abscessus abscessus*.

d Breakpoint predominantly for *M. chelonae* (to be interpreted with due limitations).

e Insufficient data to interpret. MICs: 0.12 (9%), 0.25 (23%), 0.5 (44%), 1 (20%) and 2 (4%).

**Table 2:** List of mycobacteriophages used in this study

Phage	Cluster	Reference
BPsΔ*33*HTH_HRM10	G1	Dedrick et al. [15]
Muddy	AB	
Itos	L2	
ZoeJΔ*43-45*	K2	Unpublished – Hatfull lab
FionnbharthΔ*45*Δ*47*	K4	Dedrick et al. [14]
Fred313cpmΔ*33*	A3	
D29_HRM^GD40^	A2	
phiGD20-1	MabA1	Dedrick et al. [26]
phiGD89-1	MabB	
phiT45-1	MabG	Amarh et al. 21a. https://doi.org/10.1128/MRA.00155-21.
phiT46-1	MabH	Amarh et al. 21b. https://doi.org/10.1128/MRA.01421-20.

## Data Availability

The WGS genome data are available and have been submitted to NCBI (BioProject number: PRJNA941281).
