# Supplementary material for: The problem of Mycobacterium abscessus complex: multi-drug resistance, bacteriophage susceptibility and potential healthcare transmission
Source: Clin Microbiol Infect. Author manuscript; Available in PMC 2023 Oct 18. (PMC10583746; doi:10.1016/j.cmi.2023.06.026)
Supplement: Table S1 [file NIHMS1935879-supplement-Table_S1.docx]

| **Less than 10 SNP** |  |  |  |
| --- | --- | --- | --- |
| **Strain names** |  | **#SNP** | **Notes** |
| T2753 | T2338 | 10 | Same patient A (2018 and 2019) |
| T3861 DCC1 | T1109 DCC1 | 5 | Same patient B (2018 and 2019) |
| T4702 DCC1 | T4044 DCC1 | 5 | Patient C and D (2018 and 2019) |
| T7087 DCC1 | T4044 DCC1 | 5 | Patient E and D (2018 and 2019) |
| T7087 DCC1 | T4702 DCC1 | 8 | Patient E and C (both 2018) |
| T7722 DCC1 | T4044 DCC1 | 5 | Patient F and D (2018 and 2019) |
| T7722 DCC1 | T4702 DCC1 | 7 | Patient F and C (both 2018) |
| T7722 DCC1 | T7087 DCC1 | 10 | Patient F and E (both 2018) |
|  |  |  |  |
| **Less than 20 SNP** |  |  |  |
| **Strain names** |  | **#SNP** | **Notes** |
| T2314 ND | T2183 ND | 11 | Same patient G (2018 and 2019) |
| T4848 DCC1 | T4044 DCC1 | 18 | Patient H and D (2018 and 2019) |
| T4848 DCC1 | T4702 DCC1 | 19 | Patient H and C (both 2018) |
| T7087 DCC1 | T4848 DCC1 | 20 | Patient E and H (both 2018) |
| T7221 DCC4 | T7300 DCC4 | 18 | Same patient I (2018 and 2019) |

**Table S1:** *M. abscessus* isolates with less than 10 and 20 SNP difference. They are highlighted in green if the sample was isolated from the same patient, in yellow from different patients. The DCC of each isolate is also shown**.**
