# Supplementary material for: The problem of Mycobacterium abscessus complex: multi-drug resistance, bacteriophage susceptibility and potential healthcare transmission
Source: Clin Microbiol Infect. Author manuscript; Available in PMC 2023 Oct 18. (PMC10583746; doi:10.1016/j.cmi.2023.06.026)
Supplement: Table S3 [file NIHMS1935879-supplement-Table_S3.docx]

**Table S3.** Lytically propagated spontaneously induced prophages.

| **Phage name^1^** | **Donor strain^2^** | **Recipient strains^3^** | **Predicted Cluster^4^** |
| --- | --- | --- | --- |
| phiT1615 | T1615 | T5347, GD40, GD41 | MabH |
| phiT2686B | T2686B | T5347, GD40, GD41, GD150B, GD123 | MabA1 |
| phiT5953B | T5953B | T5347, GD41 | ND |
| phiT6058 | T6058 | T5347, T2686A, T2686B, T6059, T7193B, T9538B, GD40, GD41, GD150B, GD123 | MabA1 |
| phiT6059 | T6059 | T5347, GD40, GD41, GD150B, GD123 | MabA1 |
| phiT7824 | T7824 | T5347, T7193B, GD40, GD41, GD150B, GD123 | MabA1 |
| phiT5953A | T5953A | T5347, GD40, GD150B | ND |
| phiT11795 | T11795 | T2314, T1615, T1109A, T1109B, T1777, T2686A, T2686B, T2774, T2286, T5538, T4702, T3497, T7518, T6058, T5953A, T5919, T5347, T7722, T5002, T7605, T7087, T7193A, T7193B, T7279, T8928A, T7821, T8763, T8251, T7985A, T7985B, T8582A, T9538A, T9538B, T9907, T9577, T10936, T10353, T10091, T3955, T9277A, T9277B, T545, T11795, T12216, T13007, T12193, T011471, GD40, GD41, GD150B, GD123 | ND |
| phiT2338 | T2338 | T7193B, GD123 | MabN |
| phiT3408 | T3408 | T5953A, T6059, T7193B | MabH |
| phiT4388 | T4388 | T2314, T1777, T2753, T7518, T5953A, T6059, T7193B, T7821, T7985A, T7985B, T8582A, T9538A, T9538B, T2338, T9277A, T9277B, T545, T13007, T011471, GD123 | MabN |
| phiT4702 | T4702 | T6059, T7193B | MabA1 |
| phiT545 | T545 | T6059, T7193B, T8582A | MabH |
| phiT8251 | T8251 | T7193B, T8582A, T9277A, T9277B, T545, T011471, | MabN |
| phiT8845 | T8845 | T1777 | MabC |
| phiT10269 | T10269 | T1777 | MabC |
| phiT4044 | T4044 | T9277A | MabC |
| phiT608 | T608 | T12193 | MabN |
| phiT7722 | T7722 | GD123 | MabC |
| phiT11642 | T11642 | GD123 | MabB |
| phiT5347 | T5347 | GD123 | MabB |
| phiT9277 | T9277B | GD123 | ND |
| phiT9538 | T9538 | GD123 | ND |
| phiT9875 | T9875 | GD123 | ND |
| phiT1777 | T1777 | GD123 | ND |

ND, Not Determined

^1^Derived phages are designated as prophi*donorstrainname*.

^2^The *M. abscessus* strain which the isolated prophage was released from.

^3^Strains, including additional lab isolates (indicated as GDxx), that are infected by spontaneously released particles from the donor strain or subsequently propagated phages.

^4^Predicted cluster based on PCR amplification of phage lysate using cluster-specific primers. ND, Not Determined.
