# Supplementary material for: The problem of Mycobacterium abscessus complex: multi-drug resistance, bacteriophage susceptibility and potential healthcare transmission
Source: Clin Microbiol Infect. Author manuscript; Available in PMC 2023 Oct 18. (PMC10583746; doi:10.1016/j.cmi.2023.06.026)
Supplement: Table S2 [file NIHMS1935879-supplement-Table_S2.docx]

**Table S2**. Bioinformatically predicted *M. abscessus* prophages.

| **Host Strain^1^** | **Prophage^2^** | **Prophage Cluster^3^** | **Program^4^** | **Contig^5^** | **Duplicate^6^** |
| --- | --- | --- | --- | --- | --- |
| T6814 | prophiT6814-1 | MabH | DEPhT | 8 |  |
| T9403 | prophiT9403-1 | MabC | DEPhT | 3 |  |
| T2283 | prophiT2283-1 | MabL | DEPhT | 9 |  |
| T2283 | prophiT2283-2 | MabB | DEPhT | 9 |  |
| T11960 | prophiT11960-1 | MabG | DEPhT | 10 |  |
| T11795 | prophiT11795-1 | MabL | DEPhT | 4 |  |
| T11795 | prophiT11795-2 | MabA | DEPhT | 5 |  |
| T3648 | prophiT3648-1 | MabH | DEPhT | 17 |  |
| T3648 | prophiT3648-2 | MabG | DEPhT | 22 |  |
| T7824 | prophiT7824-1 | MabC | DEPhT | 3 | prophiT9566-1 |
| T7824 | prophiT7824-2 | MabA | DEPhT | 10 |  |
| T7087 | prophiT7087-1 | MabB | DEPhT | 5 | prophiT4702-1 |
| T7087 | prophiT7087-2 | MabA | DEPhT | 11 | prophiT4044-2 |
| T2959 | prophiT2959-1 | MabH | DEPhT | 29 |  |
| T5347 | prophiT5347-1 | MabD | DEPhT | 15 |  |
| T5347 | prophiT5347-2 | MabB | DEPhT | 29 |  |
| T1377 | prophiT1377-1 | MabE | DEPhT | 25 |  |
| T1377 | prophiT1377-2 | MabL | DEPhT | 9 |  |
| T7279 | prophiT7279-1 | MabB | DEPhT | 26 |  |
| T7279 | prophiT7279-2 | MabL | DEPhT | 13 |  |
| T9577 | prophiT9577-1 | MabB | DEPhT | 4 |  |
| T9141 | prophiT9141-1 | MabJ | DEPhT | 24 |  |
| T1777 | prophiT1777-1 | MabJ | DEPhT | 8 |  |
| T5363 | prophiT5363-1 | MabB | DEPhT | 15 | prophiT5953-1 |
| T2183 | prophiT2183-1 | MabG | DEPhT | 8 |  |
| T2183 | prophiT2183-2 | MabC | DEPhT | 31 |  |
| T9553 | prophiT9553-1 | MabG | DEPhT | 1 | prophiT3408-2 |
| T9553 | prophiT9553-2 | MabA | DEPhT | 28 |  |
| T9538 | prophiT9538-1 | MabS | DEPhT | 12 |  |
| T608 | prophiT608-1 | MabN | DEPhT | 1 |  |
| T608 | prophiT608-2 | MabE | DEPhT | 4 | prophiGD04-1 |
| T5953 | prophiT5953-1 | MabB | DEPhT | 15 |  |
| T6058 | prophiT6058-1 | MabA | DEPhT | 4 |  |
| T6058 | prophiT6058-2 | MabH | DEPhT | 7 |  |
| T8538 | prophiT8538-1 | MabL | DEPhT | 7 |  |
| T12193 | prophiT12193-1 | MabB | DEPhT | 1 |  |
| T12193 | prophiT12193-2 | MabE | DEPhT | 11 |  |
| T9277 | prophiT9277-1 | MabJ | DEPhT | 19 |  |
| T3955 | prophiT3955-1 | MabB | DEPhT | 16 |  |
| T503 | prophiT503-1 | MabE | DEPhT | 6 | prophiGD04-1 |
| T8923 | prophiT8923-1 | MabH | DEPhT | 32 |  |
| T7300 | prophiT7300-1 | MabE | DEPhT | 4 | prophiT011292-2 |
| T7300 | prophiT7300-2 | MabG | DEPhT | 4 |  |
| T7300 | prophiT7300-3 | MabA | DEPhT | 7 |  |
| T8314 | prophiT8314-1 | MabS | DEPhT | 4 |  |
| T8314 | prophiT8314-2 | MabK | DEPhT | 5 |  |
| T2286 | prophiT2286-1 | MabG | DEPhT | 1 |  |
| T4388 | prophiT4388-1 | MabH | DEPhT | 6 |  |
| T4388 | prophiT4388-2 | MabN | DEPhT | 7 |  |
| T4388 | prophiT4388-3 | MabJ | DEPhT | 75 |  |
| T7193 | prophiT7193-1 | MabA | DEPhT | 6 |  |
| T2022 | prophiT2022-1 | MabL | DEPhT | 19 |  |
| T11289 | prophiT11289-1 | MabH | DEPhT | 2 |  |
| T11289 | prophiT11289-2 | MabG | DEPhT | 4 |  |
| T11289 | prophiT11289-3 | MabB | DEPhT | 6 |  |
| T6502 | prophiT6502-1 | MabL | DEPhT | 27 |  |
| T10936 | prophiT10936-1 | MabG | DEPhT | 1 |  |
| T7821 | prophiT7821-1 | MabJ | DEPhT | 21 | prophiT1777-1 |
| T11047 | prophiT11047-1 | MabN | DEPhT | 37 |  |
| T7221 | prophiT7221-1 | MabA | DEPhT | 9 |  |
| T7221 | prophiT7221-2 | MabE | DEPhT | 12 | prophiT011292-2 |
| T7221 | prophiT7221-3 | MabG | DEPhT | 23 |  |
| T8845 | prophiT8845-1 | MabC | DEPhT | 20 |  |
| T1109 | prophiT1109-1 | MabG | DEPhT | 25 |  |
| T9566 | prophiT9566-1 | MabC | DEPhT | 2 |  |
| T9566 | prophiT9566-2 | MabA | DEPhT | 5 | prophiT7824-2 |
| T7605 | prophiT7605-1 | MabL | DEPhT | 32 |  |
| T4848 | prophiT4848-1 | MabB | DEPhT | 4 | prophiT4702-1 |
| T4848 | prophiT4848-2 | MabA | DEPhT | 12 | prophiT4044-2 |
| T9765 | prophiT9765-1 | MabG | DEPhT | 5 |  |
| T9765 | prophiT9765-2 | MabE | DEPhT | 5 | prophiT011292-2 |
| T9765 | prophiT9765-3 | MabA | DEPhT | 9 |  |
| T2314 | prophiT2314-1 | MabG | DEPhT | 28 |  |
| T011292 | prophiT011292-1 | MabG | DEPhT | 11 |  |
| T011292 | prophiT011292-2 | MabE | DEPhT | 15 |  |
| T3408 | prophiT3408-1 | MabJ | DEPhT | 1 | prophiGD24-3 |
| T3408 | prophiT3408-2 | MabG | DEPhT | 5 |  |
| T3408 | prophiT3408-3 | MabA | DEPhT | 6 |  |
| T5734 | prophiT5734-1 | MabA | DEPhT | 7 |  |
| T4044 | prophiT4044-1 | MabB | DEPhT | 6 | prophiT4702-1 |
| T4044 | prophiT4044-2 | MabA | DEPhT | 12 |  |
| T3861 | prophiT3861-1 | MabG | DEPhT | 24 | prophiT1109-1 |
| T9907 | prophiT9907-1 | MabA | DEPhT | 23 |  |
| T9875 | prophiT9875-1 | Singleton | DEPhT | 2 |  |
| T9875 | prophiT9875-2 | MabO | DEPhT | 26 |  |
| T10269 | prophiT10269-1 | MabC | DEPhT | 2 |  |
| T8763 | prophiT8763-1 | MabE | DEPhT | 1 | prophiGD04-1 |
| T7722 | prophiT7722-1 | MabA | DEPhT | 4 | prophiT4044-2 |
| T7722 | prophiT7722-2 | MabB | DEPhT | 11 | prophiT4702-1 |
| T4702 | prophiT4702-1 | MabB | DEPhT | 4 |  |
| T4702 | prophiT4702-2 | MabA | DEPhT | 12 | prophiT4044-2 |
| T10091 | prophiT10091-1 | MabL | DEPhT | 26 | prophiT7279-2 |
| T7985 | prophiT7985-1 | MabA | DEPhT | 33 |  |
| T1615 | prophiT1615-1 | MabG | DEPhT | 5 |  |
| T7518 | prophiT7518-1 | MabC | DEPhT | 21 |  |
| T11960 | prophiT11960-2 | MabH | DEPhT+SPLICE | 7;35;36;1;36 |  |
| T11960 | prophiT11960-3 | MabC | DEPhT+SPLICE | 1;24;30 |  |
| T7087 | prophiT7087-3 | MabC | DEPhT+SPLICE | 1;24 |  |
| T9577 | prophiT9577-2 | MabA | DEPhT+SPLICE | 11;34;53 |  |
| T9553 | prophiT9553-3 | MabJ | DEPhT+SPLICE | 33;29 |  |
| T2286 | prophiT2286-2 | MabA | DEPhT+SPLICE | 9;35;36;35 |  |
| T11047 | prophiT11047-2 | MabA | DEPhT+SPLICE | 31;12 |  |
| T8845 | prophiT8845-2 | MabN | DEPhT+SPLICE | 42;5 |  |
| T2314 | prophiT2314-2 | MabC | DEPhT+SPLICE | 5;26;35 |  |
| T3497 | prophiT3497-1 | MabA | DEPhT+SPLICE | 18;32 |  |
| T4044 | prophiT4044-3 | MabC | DEPhT+SPLICE | 22;4 |  |
| T7722 | prophiT7722-3 | MabC | DEPhT+SPLICE | 25;6 |  |
| T4702 | prophiT4702-3 | MabC | DEPhT+SPLICE | 25;1 |  |
| T1615 | prophiT1615-2 | MabH | DEPhT+SPLICE | 6;34;2;34 |  |
| T1615 | prophiT1615-3 | MabC | DEPhT+SPLICE | 2;21;25 |  |
| T7518 | prophiT7518-2 | MabM | DEPhT+SPLICE | 21;20 |  |

^1^The *M. abscessus* strain in which the prophage was identified.

^2^Prophages are designated as prophi*strainname* with a suffix appended to distinguish different prophages in the same strain.

^3^Each prophage is assigned to a cluster as described previously (Dedrick et al., ’21)

^4^The program or combination of programs used to identify the prophages are shown.

^5^The contig(s) containing each prophage is indicated. Prophages identified by DEPhT are within a single contig and are complete. Prophages identified by DEPhT and SPLICE typically span two or more contigs and are incomplete.

^6^Duplicates of prophages with identical nucleotide sequences are indicated. Prophages reported by SPLICE are incomplete and thus have not been screened for duplication.
